# Maximizing the Sensitivity of Bilateral Inferior Petrosal Sinus Sampling

**DOI:** 10.1210/jendso/bvae094

**Published:** 2024-05-08

**Authors:** Alan Kelsall, John Newell-Price

**Affiliations:** School of Medicine and Population Health, University of Sheffield, Sheffield S10 2RX, UK; School of Medicine and Population Health, University of Sheffield, Sheffield S10 2RX, UK

**Keywords:** Cushing's, pituitary, ACTH, petrosal, diagnosis, cortisol

Commentary on: Chen S, Lyu X, Hong W, Zhang D, Zhang Y, Yang D, et al Bilateral Inferior Petrosal Sinus Sampling Without Lateralization Is Less Accurate for the Diagnosis of Cushing Disease. J Endocr Soc. 2024; 8(5):bvae056.

The diagnosis of Cushing's disease may be difficult and requires both careful clinical assessment and the selection of biochemical and imaging studies. Given the potential for nonneoplastic conditions and certain physiological states to mimic Cushing's syndrome, investigations should be driven by strong clinical suspicion.

Following confirmation of ACTH-dependent Cushing's syndrome, magnetic resonance imaging is recommended to help distinguish between Cushing's disease (CD) and ectopic ACTH syndrome (EAS), with findings of a pituitary lesion ≥ 10 mm consistent with CD. In those with a pituitary microadenoma or equivocal findings on magnetic resonance imaging, one can proceed either directly to bilateral inferior petrosal sinus sampling (BIPSS), particularly if the adenoma is <6 mm, or take a noninvasive approach involving a combination of corticotropin-releasing hormone (CRH) and desmopressin stimulation tests alongside a whole body computed tomography imaging [1].

Unfortunately, in most areas of the world, CRH is not currently available. Desmopressin has been used as an alternative ACTH-secretagogue. Peripherally administered desmopressin as a noninvasive test causes stimulation of vasopressin 1b receptors, commonly expressed in corticotropinomas, resulting in secretion of ACTH, with an overall sensitivity of 85% and specificity of 70% for the diagnosis of CD, significantly lower than that of CRH (87% and 94%, respectively) [2]. The use of desmopressin as a peripheral standalone test carries a substantial risk of incorrect diagnosis and management.

BIPSS is currently the gold-standard test in differentiating between EAS and CD [3]. A recent meta-analysis of 25 studies reported a sensitivity of 86% and specificity of 98% before stimulation using a central inferior petrosal sinus: peripheral (IPS:P) ACTH ratio > 2 and a sensitivity of 97% and specificity of 100% following CRH stimulation, with IPS:P ratio > 3 [4]. Given the lack of CRH supply, desmopressin has been shown to be an effective and safe alternative in BIPSS with 96% sensitivity and 100% specificity [5]. These sensitivity ratios have to be set against the fact that in women with ACTH-dependent disease, 90% will be due to CD, and so enhancing testing sensitivity (reducing false negatives) while maintaining specificity is of paramount importance.

Interpreting BIPSS results to predict lateralization of the microadenoma in CD has produced mixed results. Oldfield et al found 92% of patients with pituitary microadenoma confirmed at surgery had an ACTH gradient between IPS of ≥ 1.4; however, only in 68% did the gradient point toward the correct side of the adenoma [3]. Overall, lateralization is disappointing.

In this current study, Chen et al sought to determine if further analysis of lateralization could be used not for determining adenoma position but to reduce the false-negative rate of BIPSS when using desmopressin. In a retrospective study, they assessed 40 consecutive CD and 30 consecutive EAS patients in a single hospital over a 10-year period. This was done by dividing the CD group post-BIPSS into those with low lateralization (IPS:IPS ≤ 1.4, n = 11) and high lateralization (IPS:IPS > 1.4, n = 29). Digital subtraction angiography (DSA) videos were used to assess the peripituitary vascular differences between groups to see whether this may account for the observations [6].

The key finding is that the maximum IPS:P ACTH ratio was lower in the low lateralization group compared to the high lateralization group, both before (median maxIPS:P 3.17 vs 7.77, *P* = .013) and after desmopressin stimulation (median maxIPS:P 11.08 vs 18.97, *P* = .028). Using the current prestimulation cut-off IPS:P ratio of 2, the sensitivity in the low lateralization group was 54.6% [95% confidence interval (CI) 28.0-78.7%] compared to 93.1% (95% CI 78.0-98.1%) in the high lateralization group. Following desmopressin stimulation in the low lateralization group, sensitivity increased to 90% (95% CI 59.6-98.2%) but at the cost of a reduction in specificity (prestimulation 100%, 95% CI 88.7-100%; to poststimulation 76.7%, 95% CI 59.1-88.2%). The accuracy of adenoma lateralization prediction in the high lateralization group was 66%, in keeping with previous studies. Applying the findings from this discovery set to a further validation set of 220 patients with CD (without DSA data) divided into low (n = 30) and high (n = 190) lateralization results confirmed sensitivities before and after desmopressin of 50% and 76.7%, and 93.7% and 96.3%, respectively.

To investigate whether variations in anatomy contributed to the observed differences, digital subtraction angiography was performed to characterize peripituitary vasculature. This demonstrated less contrast drainage to the contralateral inferior petrosal sinus in the low lateralization group. The authors propose that the complexity of the peripituitary vasculature in the low lateralization group, with possible distribution of ACTH into other small vessels rather than the inferior petrosal sinus, could hence lead to a reduction of the measured central to peripheral (IPS:P) ratio. Additionally, the authors hypothesized that these anatomical differences could account for the higher peripheral ACTH values seen in the low lateralization group [6].

A previous retrospective study of 17 patients with confirmed CD assessed inferior petrosal sinus drainage manually by injecting contrast into the right IPS. Patients were grouped by venography findings as left or right side dominant or symmetrical. Although a small sample size, the lateralization, or absence thereof, of IPS:IPS matched the venous drainage in all patients [7].

A benefit of the current study was the use of DSA, reducing the risk of interpretive bias; however, images were selected manually and therefore until there is a standardized automated process, the use of DSA in characterizing peripituitary vasculature and aiding BIPSS interpretation is likely to show interoperator variability.

Overall, this new analysis of BIPSS may have value in clinical practice. While further prospective studies with larger sample sizes are required to confirm the findings, clinicians may cautiously use these data to aid decision-making in borderline cases, as low lateralization results are more likely to be false negative. Thus, additional care and reflection on the results of BIPSS may be needed in such cases to avoid patients being labeled as having EAS, especially when hypercortisolemia is on the milder end of the spectrum [8].

## Disclosures

None.

## References

[1] Fleseriu M, Auchus R, Bancos I, et al Consensus on diagnosis and management of Cushing’s disease: a guideline update. Lancet Diabetes Endocrinol. 2021;9(12):847‐875. 34687601 10.1016/S2213-8587(21)00235-7 PMC8743006

[2] Ceccato F, Barbot M, Mondin A, Boscaro M, Fleseriu M, Scaroni C. Dynamic testing for differential diagnosis of ACTH-dependent cushing syndrome: a systematic review and meta-analysis. J Clin Endocrinol Metab. 2023;108(5):e178‐e188. 36453141 10.1210/clinem/dgac686

[3] Oldfield EH, Doppman JL, Nieman LK, et al Petrosal sinus sampling with and without corticotropin-releasing hormone for the differential diagnosis of Cushing’s syndrome. N Engl J Med. 1991;325(13):897‐905. 1652686 10.1056/NEJM199109263251301

[4] Chen S, Chen K, Wang S, et al The optimal cut-off of BIPSS in differential diagnosis of ACTH-dependent Cushing’s syndrome: is stimulation necessary? J Clin Endocrinol Metab. 2020;105(4):e1673‐e1685. 10.1210/clinem/dgz194 31758170

[5] Valizadeh M, Ahmadi AR, Ebadinejad A, Rahmani F, Abiri B. Diagnostic accuracy of bilateral inferior petrosal sinus sampling using desmopressin or corticotropic- releasing hormone in ACTH-dependent Cushing’s syndrome: a systematic review and meta-analysis. Rev Endocr Metab Disord. 2022;23(5):881‐892. 35478451 10.1007/s11154-022-09723-y

[6] Chen S, Lyu X, Hong W, et al Bilateral inferior petrosal sinus sampling without lateralization is less accurate for the diagnosis of cushing disease. J Endocr Soc. 2024;8(5):bvae056. 10.1210/jendso/bvae056 PMC10989187 38572419

[7] Ghorbani M, Akbari H, Griessenauer CJ, et al Lateralization of inferior petrosal sinus sampling in Cushing’s disease correlates with cavernous sinus venous drainage patterns, but not tumor lateralization. Heliyon. 2020;6(10):e05299. 33134585 10.1016/j.heliyon.2020.e05299 PMC7586104

[8] Lavoillotte J, Mohammedi K, Salenave S, et al Personalized non-invasive diagnostic algorithms based on urinary free cortisol in ACTH-dependant Cushing’s syndrome. J Clin Endocrinol Metab. 2024; doi:10.1210/clinem/dgae258 38609171

